# The New York Academy of Medicine and Dr. Horace Green Again

**Published:** 1855-08

**Authors:** 


### THE NEW YORK ACADEMY OF MEDECINE AND DR. HORACE GREEN, AGAIN.

The New York Academy of Medicine and Dr. Green have furnished the text of several articles from our pen, and, perhaps, some of our indulgent readers may think that we have already devoted to the Academy and the Academecian enough of our editorial space. Catheterization of the air-passages, however, is intrinsically a subject of so much importance, and the extent to which Dr. Green claims to have carried this mode of medication, is a matter of such variance of opinion among equally competent and honest observers, and its final decision a question of such practical interest, that we deem it a duty again to place our readers *au courant* with all that relates to it.

It will be remembered that, when Dr. Green read his paper on catheterization of the air-passages, etc., to the Academy, a committee was at once appointed "to inquire into and examine" the proposed treatment. This committee consisted of Drs. Willard Parker, A. Stevens, Isaac Wood, B. F. Barker, John O. Stone, J. F. Metcalfe, and James Anderson. The committee was instructed to endeavor first, to settle the question of the passage of the tube into the trachea, and second, as far as possible, to ascertain the utility of the injections of the nitrate of silver into the lungs.

The committee met five times—twice by invitation at Dr. Green's office, and three times at Bellevue Hospital. Thirty-eight patients in the aggregate were submitted by Dr. Green and others to the test of the operation of catheterization of the air-passages, and then of the injection of nitrate of silver into these passages. The results of the series of experiments "confirm the records of history as to the practicability of the operation of catheterizing the air-passages." In eleven cases it was performed to the entire satisfaction of the committee. In these the tube was detected in the cavity of the larynx by the finger passed into the mouth, and the symptoms manifested by the patient were pathognomonic. (It is needless to remark that they were those of a foreign body in the air-passages.) In the remaining twenty-seven cases the committee had reason to believe that the tube had passed into the œsophagus. The symptoms which distinguish the passage of an instrument into the trachea and œsophagus, are thus tabulated by the committee:

| Trachea. | Œsophagus. |
| --- | --- |
| 1st. Suffusion of the face, rapidly increasing to turgesence and lividity. | 1st. Suffusion of the face slightly, rapidly subsiding, with cessation of retching and cough. |
| 2d. Great anxiety and alarm, not easily pacified. | 2d. Little anxiety, easily pacified. |
| 3d. Eyes wild, staring and overflowing with tears. | 3d. Eyes natural, slight suffusion from tears. |
| 4th. Cough violent and spasmodic. | 4th. Little or no cough. |
| 5th. Respiration greatly disturbed; inspiration loud, hoarse and stridulous, expiration attended with violent cough, like that of laryngeal phthisis, ejection of bronchial mucus through the tube, and finally free breathing through the tube. | 5th. Respiration little if at all disturbed. |
| 6th. Voice extinguished; a hoarse whisper, interpeted with difficulty. | 6th. Voice distinct, often quite natural. |
| 7th. Retching slight. | 7th. Retching and vomiting a common symptom. |

These symptoms were so unvarying and contrasted so strikingly that the committee state that they regard the rational signs as the surest criterion of the success of the operation. They were exhibited with characteristic intensity in all but a single instance, and in this syphilitic ulceration had made such ravages in the structures about the superior portion of the air-passages that it cannot be considered a fair case. In the course of their experiments the committee became satisfied that the opinion of the operator as to the course and situation of the instrument is often fallacious when he relies upon his own senses and disregards symptoms. Dr. Green himself was frequently at fault. In several of the cases detailed, while positive that he had passed the instrument into the trachea, the patients vomited through the tube.

The question, then, of the practicability of the operation is henceforth set at rest. Its facility, however, is still a matter about which opinions differ, and depends in the opinion of the committee more upon the form of the instrument used than upon the previous preparation of the patient by the frequent applications of nitrate of silver to the upper portions of the larynx—a point so much insisted upon by Dr. Green.

The committee employed three instruments in their experiments, namely: 1st, Hutching's catheter (No. 10) with a wire stilet, and curved like a common catheter; 2d, one of the same size slightly bent at its extremity, (this is the instrument used by Dr. G. in practice), and 3d, a sponge-armed probang, the sponge having a diameter of from one-half to three-fourths of an inch.

Of thirteen trials of the tube with the large curve, eight succeeded; of thirty-eight trials of the tube with the small curve, three succeeded; *and of eighteen trials of the sponge probang, all failed.*

Now, this seems to us very strange. Dr. Green asserts "that this method of medicating the larynx and trachea is accomplished with much ease and ordinarily with great certainty," and "that the sponge-armed probang . . . . . . has been in a thousand instances thrust down between and beyond the vocal chords, and has been carried not only through the trachea and bifurcations, but . . . . . . has been passed *at will* into the right or left bronchial division." The committee, composed of gentlemen, and all of more or less reputation for professional skill, with Willard Parker at its head, found, after a series of carefully conducted experiments, many of them upon patients who had been prepared for catheterizing by Dr. Green himself, that with the tube of the most approved curve the number of failures amounts to thirty-eight per cent.; that the tube with which Dr. Green is accustomed to throw injections into the air-passages failed of entering the trachea in ninety-two per cent. of the trials; and that the sponge-probang failed in every instance to enter the trachea.

The committee, not being satisfied of the practicability of passing a tube into tubercular cavities in the lungs, dismiss without remark the question of the probable utility of injections into such cavities; and very properly it seems to us.

Injections of nitrate of silver were thrown into the air-passages in three patients. Two did not again come under the notice of the committee though it is elsewhere stated that both individuals were benefitted by the treatment. The third case terminated fatally, and the committee believe that the injection was the immediate cause of death. We confess, however, that we do not altogether agree with the committee in this conclusion. The evidence on which they base their opinion is not as satisfactory as it might be. We may conscientiously believe that the death of the patient is chargeable to the injection, but, we repeat, that we do not think that the inference necessarily flows from the testimony adduced.

The committee close their report with the following conclusions, signed by three of the committee—Parker, Stone, and Wood, and subsequently by Metcalfe:

1st. Catheterism of the air-passages dates its history from the time of Hippocrates.

2d. The best evidences of the passage of an instrument into the air-passages, are the rational signs.

3d. The facility of the operation depends upon the kind of instrument used; the tube having a large curve being best, and the sponge probang least adapted to enter the trachea.

4th. That there is no reliable evidence, in the opinion of your committee, that the *sponge probang* has been passed through and beyond the vocal chords.

5th. That there is no positive evidence that an instrument can be passed at will into the right or left bronchial divisions.

6th. That in the great majority of instances where injections are supposed to have been thrown into the lungs, through a tube, they have passed directly into the stomach.

7th. That as regards the utility of injections of nitrate of silver into the lungs, the facts thus far developed, in the experiments of your committee, lead them to regard the operation as one fraught with danger, as well as difficulty.

Dr. Anderson did not think that the second division had received "that investigation which would enable them intelligibly to report upon it," and so declined attaching his signature to this division of the report. He signed however the first.

Dr. Stevens made a separate report: "As regards the question,' he remarks, "of the practicability of the introduction of the probang into the trachea, he did not see enough to enable him to consider the practicability of such introduction as a proved fact. But of the practicability of passing a catheter into the trachea, he was confirmed in his previous belief, that it admits of no question whatsoever." He moreover regarded it as proved that injections of the air-passages were "quite practicable with ordinary skill." The safety and therapeutic value of such injections, however, he did not think determined.

"In regard to the question of the practicability of directing the solution to a particular part of the bronchial tubes, or into any cavity connected with them, at the pleasure of the operator, he has seen nothing to lead him to an affirmative answer."

Here, then, are four gentlemen who sign an out-and-out report, one who subscribes to its larger part, and another who signs pretty nearly the whole—in all six. The name of the seventh member of the committee, Dr. B. Fordyce Barker, Dr. Green's colleague, is alone wanting. This appears attached to a minority report of considerable length and more feeling. We do not propose to notice the latter element of the report, except very briefly. Dr. Barker would have done himself more credit and the cause of his colleague, Dr. Green, better service, if all this portion of his report had been omitted. The profession are not likely to believe that Willard Parker and his fellow-committeemen are any more influenced by personal feeling in their report than Dr. Barker is in his. The experiments on which they base their conclusions are all detailed with such minuteness that a mere beginner can tell at a single glance what are their legitimate teachings, and whether they are reconcilable or in keeping with Dr. Green's claims, without the aid of a key from Dr. Barker.

Hear-say is not evidence in a case like the present—indeed individual experience and individual belief are hardly admissible. The testimony of Dr. Barker's strongest witness is to be received with many grains of allowance. Dr. Watson, of New York, and Dr. Watson, of Glasgow, may both very honestly believe that they have passed the sponge probang into the trachea between the vocal chords, and yet be mistaken. How can this be doubted when after the originator of the mode of practice in question, Dr. Horace Green himself, while in several instances positive that he had successfully carried Hutching's catheter into the trachea, the patients vomited through the tube, and thus demonstrated his error.

But we will not prolong our remarks. We do not wish to be considered partisans in this discussion among the New York Academecians—we are mere lookers on and simply desirous of furnishing our readers with the latest tntelligence from "the seat of war." Two more widely different documents than the majority and minority reports, could hardly have been based upon the same data. We have given the gist of the former—the following is the conclusion of the latter:

1st. Direct medication of the lungs by means of catheterism of the air-tubes, was first proposed and carried into effect by our associate member, Dr. Horace Green.

2d. The operation may be performed by the dexterous surgeon with ease and facility, and with perfect safety to the patient.

3d. The results of this method of treatment, whether it has been employed in bronchial affections or in the commencement of tuberculosis, have already afforded the most gratifying indications that practical medicine will be greatly advanced by this discovery.

We confess that, to our minds, neither report is satisfactory, nor do the conclusions of either appear to us wholly correct. We think that the report of the majority does not give Dr. Green the full merit to which his labors in this direction entitle him, and we are on the other hand sure that Dr. Barker claims for his friend and colleague more than facts thus far warrant. D. W. Y.

